# Nutritional and Functional Potential of Carob Syrup *Versus* Date and Maple Syrups

**DOI:** 10.17113/ftb.60.02.22.7419

**Published:** 2022-06

**Authors:** Imad Toufeili, Marwa Itani, Mona Zeidan, Osama Al Yamani, Samer Kharroubi

**Affiliations:** 1 Department of Nutrition and Food Sciences, American University of Beirut, Riad El Solh 1107 2020, Beirut, Lebanon; 2 Toxicology Research and Training Center, Faculty of Health Sciences, American University of Science and Technology, Beirut, Lebanon

**Keywords:** syrup, *Ceratonia siliqua* L., nutritional content, phenolic profile, antioxidant capacity, hydroxymethylfurfural

## Abstract

**Research background:**

The carob tree (*Ceratonia siliqua* L.) is grown primarily for its seeds that are utilized in the production of the highly prized locust bean gum. The material left after the separation of seeds from the pods is utilized in the production of a range of traditional products including carob syrup, usually in cottage-type industries. The international market penetration of carob syrups is rather limited and, accordingly, scant information exists on their composition and phytochemical properties compared to mainstream syrups. The present study aims to determine key chemical parameters, phenolic profiles and antioxidant properties of carob syrups and benchmark these against those of date and maple syrups.

**Experimental approach:**

Carob syrups were prepared from 19 accessions of the carob, under laboratory conditions, by a similar procedure to those practiced by small-scale producers. The pH, browning index, the content of proteins, minerals, hydroxymethylfurfural, sugar composition, total phenols, antioxidant capacity and phenolic profiles of the produced syrups along with branded samples of date and maple syrups were analyzed.

**Results and conclusions:**

The pH and sugar composition of the carob syrups were comparable to those of date and maple syrups. In general, the carob syrups contained more proteins, minerals, phenolic acids, flavonoids and total phenols, and exhibited higher antioxidant capacity than the date and maple syrups. The carob syrups exhibited excessive browning and contained more, or comparable content of hydroxymethylfurfural, than the date and maple syrups. The data indicate that carob syrups provide more nutrients and possess superior antioxidant potential to date and maple syrups. The high contents of the carcinogenic hydroxymethylfurfural of the carob syrups warrant milder heating regimens in the concentration step during production.

**Novelty and scientific contribution:**

In contrast to studies based on commercial and/or homemade syrups, this work utilized a relatively large number of laboratory-prepared samples for creating a robust database for carob syrup. The results indicated that carob syrups possess superior health promotion and disease prevention effects than the widely traded date and maple syrups. In addition to their potential positive contribution to public health, carob syrups have been shown to be promising candidates for bolstering the economic returns of farmers in carob-producing countries.

## INTRODUCTION

Fruit- and tree sap-based syrups have been used for millennia as sweeteners in local cuisines worldwide. In addition to providing sweetness, fruit and tree sap syrups contain proteins, minerals, vitamins and a range of phytochemicals possessing antioxidant activity (*1*). Because of their superior health properties, food product developers are increasingly using these syrups as sugar substitutes to satisfy the demands of the health-conscious consumers for safer and more natural foods (*2*). Tree sap syrups are made by tapping the trunks of endemic trees, collecting the exuding sap and concentrating the sap into a thick syrup (*3*). Fruit syrups are usually made by heating fruit juices to different levels of total solids until the target consistencies are attained (*2*). The heating step during the making of syrups increases viscosity, generates the brown colour and develops the unique flavour profiles of the products (*4*). In addition to their functionality as sweeteners, syrups are used in the food industry to add viscosity, impart brown colour and desirable flavours, and mask bitterness in a range of food products (*4*). Amongst the tree sap syrups, maple syrup is the leading one with a forecasted global market value of $1.7 billion in 2023 (*5*). Data on the market value of fruit syrups are difficult to locate. However, inferences about the size/market penetration of fruit syrups can be made from the production statistics of the fruit, established practices of syrup production and the availability of literature on the properties and uses of the syrup. To this end, the annual production of dates has been reported at 8.9 million tonnes in 2018 (*6*), with syrup production being routinely practiced in date-growing countries (*7*) and frequently used as a sugar substitute in the formulation of foods (*2*, *5*).

Maple syrup exhibits interesting health properties including more favourable metabolic responses than those generated after ingestion of refined sugar (*8*) and antioxidant, anticancer and antimicrobial activities (*9*). Broadly similar health properties have been ascribed to date syrup with reported antioxidant and antimicrobial activities (*10*).

In the Middle East and North Africa, the carob tree (*Ceratonia siliqua* L.) is widely cultivated due to its adaptability to harsh environmental conditions and the ability to grow on marginally productive lands with low to medium rainfall (250-500 mm/year) (*11*). The carob tree bears pod-shaped fruits made up of a fleshy pulp that envelops several seeds. The seeds are rich in galactomannans and are commercially utilized in the production of locust bean gum. The carob pulp contains appreciable amounts of sugars (chiefly sucrose, glucose and fructose), dietary fibre and polyphenols, and also some proteins and a range of minerals and vitamins (*11*). It is utilized in the preparation of a range of traditional foods including carob syrup, which is extensively used as a sweetener in many parts of the world (*12*). In addition to its sweetening functionality, carob syrup has been shown to possess antioxidant activity (*13*) and superior anti-inflammatory and antimutagenic activities to cane, grape and sorghum syrups (*14*). Despite its long history of use as a sweetener and its potentially valuable health-promoting properties, scant data are available on the physicochemical and radical-scavenging properties of carob syrup. Recently, the physicochemical properties of homemade carob syrup have been reported (*15*). However, there is a dearth of information on the physicochemical properties of syrups prepared from different carob accessions under laboratory conditions. Still, no studies have attempted to compare, under the same test conditions, the antioxidant potential of carob syrup to leading tree sap and fruit syrups, *viz*. maple and date syrups. Creating a database on the physicochemical parameters and potential health effects of traditional food products and benchmarking them against known/recognized commodities within the product category are pivotal for their valorization, including possible recognition as Protected Designation of Origin (PDO) (*16*), and positioning in the global food market. Within this framework, carob syrups were prepared, under laboratory conditions, from 19 carob accessions indigenous to Lebanon, and their physicochemical parameters, antioxidant capacity and phenolic profiles were determined and compared to those of commercial maple and date syrups.

## MATERIALS AND METHODS

### Materials

Potassium hexacyanoferrate(III) trihydrate (Carrez I), zinc acetate dihydrate (Carrez II reagent kit), ammonium molybdate, ammonium trioxovanadate(V), acetonitrile, water, sodium metabisulfite, sodium bisulfite, sodium hydroxide, glucose, fructose, sucrose, Folin-Ciocalteu reagent, iron(III) chloride (hexahydrate), TPTZ (2,4,6-tri(2-pyridyl)-*s*-triazine), sodium acetate trihydrate, acetic acid, Trolox (6-hydroxy-2,5,7,8-tetramethylchromane-2-carboxylic acid), ABTS (2,2’-azino-bis(3-ethylbenzthiazoline-6-sulfonic acid), DPPH (2,2-diphenyl-1-picrylhydrazyl), potassium persulfate, gallic acid, *p*-coumaric acid, caffeic acid, *t*-cinnamic acid, syringic acid, catechin, epigallocatechin-3-gallate, quercetin and the atomic absorption standards (Ca, Na, Mg and K) were obtained from Sigma-Aldrich, Merck (Gillingham, Dorset, UK). Sodium carbonate, iron(II) sulfate, sodium thiosulfate and mercuric oxide were purchased from Merck KGaA (Darmstadt, Germany), hydrochloric acid (37%), sulfuric acid (95%) and methanol were procured from VWR International (Lutterworth, Leicestershire, UK). Light (amber colour grade A; Kirkland Signature, USA) and dark (dark colour grade A; Member’s Mark, USA) maple syrups, and date syrup (Alwadi Al Akhdar; Beirut, Lebanon) were procured from the local market.

Nineteen carob accessions, growing in the different regions of Lebanon, were used in the preparation of carob syrups. The accessions grew under diverse climatic conditions ranging in elevation between 16 and 654 m, precipitation between 491 and 1038 mm, and average temperatures between 17 and 22 °C (*17*). The samples were sorted by removing damaged pods and then washed with distilled water to remove adhering impurities. The pods were left to dry at room temperature (~25 °C) and were then placed in cloth bags and stored at room temperature until use.

### Morphological parameters of carob pods

The pod length (cm), width (cm), thickness (cm), mass (g) and the number of seeds/pod were measured on 10 randomly selected pods as described by Naghmouchi *et al*. (*18*) and are presented in Table S1.

### Preparation of carob syrups

**Fig. S1:**
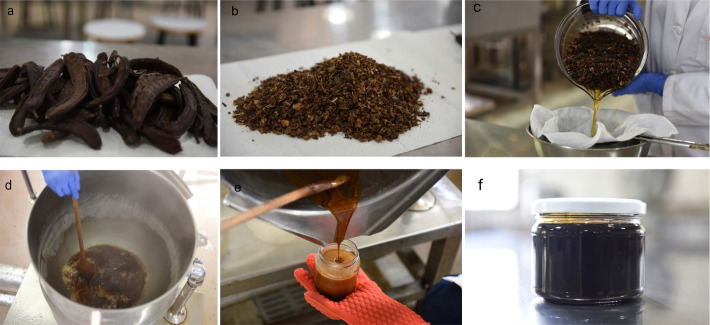
Preparation of carob syrup: a) carob pods, b) coarsely ground carob kibbles, c) filtering the carob extract, d) heat concentration of the carob extract, e) pouring the hot syrup into glass jars, and f) carob syrup

The syrups were prepared according to commercial practices followed in the production of carob syrup. The carob pods were deseeded and the coarsely ground kibbles were soaked in distilled water (1:3 *m/V*) at room temperature for 24 h and the resulting liquor was passed through a cheesecloth to remove suspended materials and then boiled in a steam-jacketed kettle until total solids of ~78-80 g/100 g were reached. The hot syrups were placed in glass jars, cooled promptly in running water (Fig. S1) and stored at 4 °C until use.

### Chemical and physicochemical analyses

Spectrophotometric analyses were performed with an Evolution 300 UV-VIS spectrophotometer (Thermo Fisher Scientific, Loughborough, UK) using Suprasil quartz cuvettes (Mettler-Toledo Ltd., Leicester, UK). All analyses were performed in triplicate and results were reported on a dry mass basis.

#### Determination of total soluble solids, pH, moisture, protein, 5-hydroxymethylfurfural and browning index

Total soluble solids (TSS), pH, and 5-hydroxymethylfurfural (HMF) were determined according to the International Honey Commission (*19*). The TSS were determined at room temperature by placing enough syrup to evenly cover the prism of an Abbe refractometer (Bellingham + Stanley, Kent, UK) and reading the TSS in ˚Bx which represents mass fraction of sucrose (1 ˚Bx=1 g sucrose per 100 g solution). The pH values of the syrups were determined at room temperature in solutions of the samples (3 g in 15 mL of distilled water) with a pH meter (SevenCompact PH/Ion meter S220; Mettler-Toledo AG, Schwerzenbach, Switzerland). The mass fraction of HMF was determined colorimetrically as per the procedure of White by treating a solution of the syrup (2.5 g in 15 mL of distilled water) with both Carrez clarification reagent kit, Carrez I (0.25 mL) and Carrez II (0.25 mL) solutions and making up to 25 mL with distilled water (*19*). The solutions were filtered and aliquots of the filtrate (2 mL) were treated with water (2 mL) or 0.2% sodium metabisulfite solution (2 mL), and the absorbance was read at 284 and 336 nm. The HMF mass fractions in the syrups were expressed in mg/kg.

Moisture and protein (N×6.25) were determined according to AOAC methods 925.45 (*20*) and 955.04 (*21*), respectively. Moisture was determined by mixing a diluted sample of the syrup (1.2-1.5 g in ~10 mL water) with acid-washed sand, heating on a steam bath (Labotec 402; Cape Town, South Africa) for 20-30 min, and then at 100 °C to a constant mass (~3-4 h). Proteins were determined by treating the syrup (~2 g) with HgO (0.7 g), anhydrous Na_2_SO_4_ (15 g) and concentrated H_2_SO_4_ (25 mL) and boiling until a clear green liquid was obtained (~1.5 h). After cooling to room temperature, the contents of the flask were diluted with distilled water (200 mL), treated with Na_2_S_2_O_3_ (25 mL, 0.3 M) and layered with concentrated NaOH (35 mL, 11 M). The NH_3_ in the flask was distilled into 0.1 M HCl and the excess HCl was titrated with 0.1 M NaOH.

The browning index (BI) was determined by measuring the absorbance of appropriate dilutions of the samples at 420 nm and converting it to the absorbance of the original sample (*22*).

#### Determination of minerals

For the determination of Na, Mg, Ca and K, the syrup (~0.5 g) was treated with concentrated HCl (15 mL) and heated at 200 °C for 30 min in a microwave digestion system (Ethos Up; Milestone, Sorisole, Italy). After cooling to room temperature, the digest was diluted to 50 mL with deionized water, and Na, Mg, Ca and K were measured by atomic absorption spectroscopy (Solaar S4 with ASX-510 autosampler; Thermo Fisher Scientific) and the concentration was determined using standard curves prepared according to AOAC method 984.27 (*23*). P was measured colorimetrically by dry ashing the syrup (~2 g) at 550 °C for 16 h, dissolving the ash in concentrated HCl (2 mL) and heating to dryness. The resulting residue was dissolved by heating in distilled water (10 mL) and the solution was filtered and made up to 50 mL with distilled water. Aliquots (5 mL) of the solution were treated with concentrated HCl and ammonium molybdate-ammonium metavanadate. The absorbance of the resulting yellow-coloured solution was measured at 400 nm and the concentration was determined using a standard curve prepared with known concentrations of phosphorus (0–50 µg/mL) (*24*).

#### Determination of sugars

Mass fractions of glucose, fructose and sucrose in the syrups were determined according to Fidan *et al.* (*25*) with some modifications. Syrup samples (~1 g) were dispersed in deionized water (25 mL), sonicated at 30 °C for 30 min and then filtered and stored at  -18 °C until analysis. The mass fractions of sucrose, fructose and glucose in the extracts were measured with HPLC (LC-10A; Shimadzu, Kyoto, Japan) using a Telos NH_2_ column (5 µm, 25 cm×4.6 mm; Kinesis Scientific Experts, Redland Bay, Australia), Telos NH_2_ guard column (5 µm, 1 cm×4.6 mm), refractive index detector, and *φ*(acetonitrile, water)=70% as mobile phase. The quantification of the sugars was made using calibration curves constructed with standard solutions of sucrose, glucose and fructose.

### Determination of total phenolic content

The phenols were extracted by shaking the syrup (4 g) with *φ*(methanol, water)=50% (10 mL) in a shaking water bath (GFL Shaking Water Bath 1092; Gesellschaft fϋr Labortechnik mbH, Burgwedel, Germany) for 30 min (*26*). The solution was filtered and the filtrate was kept at -80 °C until use.

The total phenolic content (TPC) was determined according to Singleton *et al*. (*27*). An aliquot of the extract (1 mL) was mixed with 5 mL *φ*(Folin-Ciocalteu reagent, water)=50% and 20% Na_2_CO_3_ (4 mL), vortexed, incubated at room temperature in the dark for 1 h and the absorbance of the solution was measured at 765 nm. TPC was expressed in mg gallic acid equivalents (GAE) per 100 g syrup and concentration was determined using a standard curve prepared with known concentrations of gallic acid (0–200 mg/L).

### Antioxidant capacity determination by ferric reducing antioxidant power

The ferric reducing antioxidant power (FRAP) assay was carried out according to Benzie and Strain (*28*) with slight modifications. An aliquot of the extract (100 µL) was mixed with distilled water (900 µL), FRAP reagent (2 mL) and incubated at 37 °C for 30 min. The absorbance was measured at 593 nm against a blank (1 mL water with 2 mL FRAP reagent). FRAP was expressed in µM Fe(II) per 100 g syrup and concentration was determined using a calibration curve constructed with aqueous solutions of Fe(II) sulfate (0−100 µM).

### Antioxidant capacity determined by Trolox equivalent antioxidant capacity

The Trolox equivalent antioxidant capacity (TEAC) assay was performed as described by Fu *et al*. (*29*) with slight modifications. An aliquot of the extract (100 µL) was added to the ABTS^+•^ solution (3.8 mL) and kept in the dark at room temperature for 30 min. The absorbance was measured at 734 nm against a blank containing methanol, and TEAC was expressed in mmol Trolox equivalents (TE) per 100 g syrup. Concentration was determined using a standard curve prepared with known concentrations of Trolox (25−500 µM).

### Antioxidant capacity determined by the DPPH method

The DPPH assay was performed as per Dhaouadi *et al* (*13*). An aliquot of the syrup extract (or a dilution therefrom) or ascorbic acid standard solution (50 µL) was added at different concentrations (0–600 mg/L) to 60 µM DPPH in methanol (1950 µL) and incubated in the dark at room temperature for 30 min. The absorbance was measured at 515 nm against a blank containing the same amount of DPPH˙ solution and 50 µL of distilled water. The DPPH˙ inhibition (in %) was calculated using the following equation:



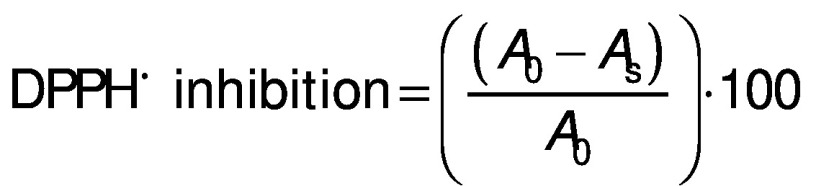



where *A*_0_ is the absorbance of the blank sample and *A*_s_ is the absorbance of the tested solution (syrup extract or ascorbic acid solution).

The results were expressed as the extract concentration providing 50% inhibition (IC_50_) in mg extract per L as determined from the plot of the absorbance *vs* extract concentration. The IC_50_ values of the syrups were also compared to the IC_50_ of known ascorbic acid solutions determined under the same conditions.

#### Identification and quantification of phenolic composition by LC-MS/MS analysis

Individual phenols were quantified with liquid chromatography-tandem mass spectrometry (LC-MS/MS) system composed of electrospray ionization (ESI) MS/MS ABI 4000 Sciex® (Toronto, Canada) coupled with liquid chromatography station comprising a quaternary pump (LC-20AD-LPG-20), autosampler (SIL-20A), column oven (CTO-20AC) and Vario preparative octadecyl silica (VP-ODS) column (150 mm×2 mm i.d., 5 µm) (Shimadzu®, Kyoto, Japan). The mobile phase comprised A: deionized water with 0.1% acetic acid, and B: 0.1% acetic acid in acetonitrile, and the gradient program was 0−4 min 85% A and 15% B, 4.01−5.00 min from 15% B to 50% B, 5.01−8.00 min 50% A and 50% B, and finally 8.01−12.00 min 85% A and 15% B. The flow rate of the mobile phase was 0.2 mL/min, the injection volume was 10 µL and the column temperature was set at 30 °C. The ESI parameters were 206 kPa for the nebulizer gas pressure, 172.4 kPa for the drying gas pressure and 400 °C for the ion source temperature. Standards of caffeic, *t*-cinnamic, *p*-coumaric, gallic and syringic acid, catechin, epigallocatechin gallate and quercetin were used in the quantification of phenols in the samples. Apart from catechin and syringic acid, which were analyzed in the negative mode with a needle voltage of -4500 V, data for the phenols were acquired in the positive mode and a needle voltage of +5500 V. **Statistical analysis**

Descriptive statistics was performed and presented to summarize the study variables of interest as mean values and standard deviations. Values of the measured parameters were subjected to one-way analysis of variance (ANOVA) and the mean values were separated by Duncan’s multiple range test when F-values were significant. All reported p-values were based on two-sided tests and were compared with a significance level of 5%. The Statistical Package for Social Sciences (SPSS) v. 25.0 for Windows (*30*) was used to analyze the data.

## RESULTS AND DISCUSSION

### Physicochemical parameters, browning index, protein and HMF mass fractions of carob, date and maple syrups

While 89.7% of the carob syrups had higher soluble solids (p<0.05) than the date syrup, all carob syrups had higher TSS (p<0.05) than the maple syrup (Table 1). Further, the TSS of carob syrups prepared in the present work were higher than those of commercial syrups from Tunisia and Turkey, which are marketed at 73−75 and 66.6−73.7 g/100 g, respectively (*15*) and those of date and maple syrup reported at 75 (*7*) and 67.1–67.4 g/100 g (*9*), respectively. In addition to being set by national standards, the TSS contents are the chief determinants of fruit and tree sap syrup viscosity/thickness which, expectedly, reflects broad national preferences.

**Table 1:** Total soluble solids (TSS), proteins, browning index, pH and hydroxymethylfurfural (HMF) of carob, maple and date syrups

Carob accession	*w*(TSS)/(g/100 g)	*w*(protein)/(g/100 g)	Browning index	pH	*w*(HMF)/ (mg/kg)
Akkar	(75.3±0.8)^b^	(2.7±0.0)^h^	(73.1±3.1)^ef^	(4.9±0.0)^jkl^	(1712.1±0.3)^gh^
Selaata	(79.8±0.3)^hi^	(2.50±0.01)^g^	(68.3±1.1)^de^	(4.93±0.02)^kl^	(13515±426^fg^
Wadi El Hojeir	(77.0±2.6)^cd^	(1.83±0.02)^e^	(87.8±0.2)^g^	(4.72±0.01)^h^	(1813±186)^h^
Batroun	(78.3±0.5)^efg^	(1.91±0.02)^e^	(45.8±2.1)^c^	(4.97±0.01)^kl^	(881±94)^cdef^
Maaroub	(78.7±0.4)^fgh^	(1.85±0.03)^e^	(31.3±1.0)^b^	(4.93±0.08)^kl^	(610±242)^bc^
Bourjin	(78.5±0.3)^fg^	(1.75±0.01)^f^	(88.9±1.8)^g^	(4.83±0.02)^ij^	(1778±265)^gh^
Marjayoun	(76.9±0.1)^cd^	(1.77±0.00)^e^	(49.0±1.1)^c^	(5.17±0.04)^m^	(779±6)^bcd^
AUB1	(77.6±0.1)^def^	(2.14±0.01)^f^	(49.1±1.4)^c^	(4.68±0.01)^gh^	(1271±303)^ef^
AUB2	(78.40±0.30)^fg^	(4.40±0.06)^k^	(85.5±10.5)^fg^	(4.88±0.02)^jk^	(1046±27)^cdef^
AUB3	(80.18±0.08)^i^	(1.81±0.02)^e^	(56.7±8.7)^cd^	(4.90±0.13)^jkl^	(814±117)^bcde^
AUB4	(79.43±0.06)^ghi^	(3.2±0.3)^j^	(57.9±1.2)^g^	(4.45±0.01)^d^	(519±210)^h^
AUB6	(80.3±0.2)^i^	(2.39±0.06)^g^	(49.8±2.1)^c^	(4.75±0.05)^hi^	(874±50)^bcde^
AUB7	(76.3±0.3)^bc^	(2.94±0.07)^i^	(52.4±4.0)^c^	(4.56±0.00)^ef^	(1103±352)^def^
AUB8	(78.8±0.2)^gh^	(0.98±0.02)^b^	(32.4±3.3)^b^	(5.0±0.1)^l^	(711±91)^bcd^
AUB9	(78.5±0.4)^fg^	(4.82±0.01)^l^	(86.9±12.1)^cd^	(4.37±0.01)^c^	(2169±514)^b^
AUB10	(78.9±0.1)^gh^	(1.35±0.02)^c^	(88.1±0.6)^g^	(4.62±0.04)^fg^	(1784±172)^gh^
AUB11	(78.8±0.2)^gh^	(2.5±0.1)^g^	(32.1±6.9)^b^	(4.73±0.02)^h^	(1121±302)^def^
AUB12	(80.3±0.3)^i^	(2.22±0.01)^f^	(109.9±16.6)^h^	(4.25±0.00)^b^	(4049±182)^i^
AUB14	(77.25±0.05)^cde^	(2.97±0.02)^i^	(127.3±5.4)^i^	(4.49±0.01)^de^	(1810±250)^h^
Date syrup	(75.50±0.00)^b^	(1.55±0.07)^d^	(98.0±23.4)^gh^	(4.01±0.03)^a^	(1987±283)^h^
Amber maple syrup	(66.6±0.1)^a^	(0.08±0.01)^a^	(3.9±0.2)^a^	(6.00±0.06)^n^	(66.2±3.0)^a^
Dark maple syrup	(66.9±0.1)^a^	(0.16±0.00)^a^	(4.1±0.2)^a^	(6.60±0.06)^o^	(72.5±2.2)^a^
Range of carob syrups	75.3−80.3	1.0−4.8	31.3−127.3	4.3−5.2	520−4049
Mean±S.D. of carob syrups	78.4±1.4	2.5±0.9	67.2±27.2	4.7±0.2	1379±809

All mass fractions were determined on dry mass basis. AUB1 to AUB14=American University of Beirut. Values with different letters in superscript in the same column are significantly different (p<0.05) according to Duncan’s multiple range test

All carob syrups had higher and lower pH (p<0.05) than date and maple syrups, respectively (Table 1). The pH of fruit and tree sap syrups depends on the content of organic acids and minerals in the sap/juice, as well as microbial contamination during processing and storage (*7*). The pH values of date and maple syrups were close to those reported at 4.2 (*31*) and 6.7−7.1 (*9*), respectively. Furthermore, the pH of carob syrup samples was similar to those of samples marketed in Tunisia and Turkey with ranges of 4.4-5.4 (*15*).

All carob syrups contained more proteins (p<0.05) than maple syrup, while 89.7% of the carob syrups had higher protein mass fractions (p<0.05) than the date syrup sample (Table 1). These findings indicate that carob syrup provides more proteins for human nutrition than date and maple syrups. The protein mass fraction of the date syrup sample was close to that of commercial samples at 1.14−1.45 g/100 g (*32*), while the maple syrup samples contained fewer proteins than reported at 0.37 g/100 g (*33*).

The maple syrup samples browned the least (p<0.05), as measured by the BI, when compared to the other syrups (Table 1). Furthermore, the carob syrups browned less than date syrup, as reflected by the lower BI (p<0.05) of 89.5% samples and the BI values reported for 6 homemade carob syrup samples from Tunisia at a mean BI value of 34 (*15*) (Table 1). The BI reflects the content of melanoidins (*34*) produced through the Maillard reaction between sugars and amino compounds and caramelization of sugars upon heating and subsequent storage. The low degree of browning observed in the maple syrup samples, as compared to the other syrups, could be attributed to their markedly lower protein content (p<0.05), higher pH (p<0.05), lower content of reducing sugars (p<0.05) (Table 2) and mild conditions for concentrating the maple sap including heating conditions needed to develop the typical colour and flavour of the finished product (*34*, *35*). The lower degree of browning of the carob syrup than of date syrup might have resulted from their lower mass fractions of reducing sugars (p<0.05) (Table 2) and higher pH (p<0.05), which limits the inversion of sucrose during the heat concentration step.

**Table 2:** Fructose, glucose and sucrose contents on dry mass basis of carob, maple and date syrups

Carob accession	*w*/(g/100 g)		
Fructose	Glucose	Sucrose
Akkar	(12.1±1.0)^b^	(7.0±0.8)^bc^	(62.0±1.7)^hi^
Selaata	(17.7±1.0)^cd^	(5.9±0.8)^b^	(59.3±3.0)^gh^
Wadi El Hojeir	(21.7±1.4)^fgh^	(10.7±0.9)^gh^	(59.3±3.0)^gh^
Batroun	(18.8±2.5)^de^	(10.0±1.5)^fgh^	(74.4±9.8)^k^
Maaroub	(16.8±0.7)^cd^	(8.3±0.6)^cdef^	(65.2±3.8)^hij^
Bourjin	(13.0±1.2)^b^	(7.1±1.0)^bcd^	(61.9±6.0)^hi^
Marjayoun	(15.7±0.7)^c^	(5.7±0.2)^b^	(67.3±1.0)^ijk^
AUB1	(23.6±2.2)^hi^	(12.7±1.5)^i^	(53.7±4.9)^fg^
AUB2	(17.8±1.5)^cd^	(9.4±1.1)^efgh^	(49.9±4.0)^ef^
AUB3	(19.3±2.2)^def^	(10.8±1.3)^gh^	(62.2±8.0)^hij^
AUB4	(19.1±0.5)^j^	(10.6±2.0)^k^	(39.7±1.1)^d^
AUB6	(23.0±0.7)^gh^	(13.2±0.8)^i^	(43.4±2.2)^de^
AUB7	(25.4±1.7)^i^	(16.9±1.0)^j^	(58.1±4.0)^gh^
AUB8	(16.2±1.1)^c^	(8.0±0.5)^cde^	(71.3±5.3)^jk^
AUB9	(39.8±0.9)^def^	(25.6±0.4)^gh^	(5.4±0.3)^a^
AUB10	(20.8±1.6)^efg^	(11.5±0.7)^hi^	(42.7±3.2)^de^
AUB11	(19.2±1.2)^def^	(8.9±1.2)^defg^	(71.0±4.5)^jk^
AUB12	(21.1±2.6)^efg^	(9.4±0.2)^efgh^	(31.7±3.9)^c^
AUB14	(37.6±0.5)^j^	(24.9±2.8)^k^	(24.5±4.3)^b^
Date syrup	(43.2±0.8)^k^	(42.9±0.8)^l^	(3.7±0.8)^a^
Amber maple syrup	(0.34±0.00)^a^	(0.00±0.00)^a^	(95.0±1.7)^l^
Dark maple syrup	(0.35±0.00)^a^	(0.35±0.00)^a^	(96.5±4.2)^l^
Range of carob samples	12.1−39.8	5.7−25.6	5.4−74.4
Mean±S.D. of carob syrups	21.0±7.1	11.4±5.6	52.9±17.9

Values with different letters in superscript in the same column are significantly different (p<0.05) according to Duncan’s multiple range test. AUB1 to AUB14=American University of Beirut

The maple and date syrup samples had the lowest and highest average levels of HMF, respectively. Furthermore, while only one sample did not differ in HMF content (p>0.05), the other 18 carob syrups contained more HMF (p<0.05) than the maple syrup samples. Only one sample contained more HMF (p<0.05), while the other carob syrup samples either did not show differences (p>0.05) or contained less HMF than the date syrup sample (Table 1). Hydroxymethylfurfural is formed through the dehydration reactions that take place during the caramelization of sugars and their heating in the presence of amino compounds in the Maillard reaction (*34*). While the formation of HMF during caramelization of sugars is independent of the confounding effects of amino compounds, the determination of the precise contribution of caramelization and the Maillard reaction to the formation of HMF in heated syrup containing sugar and amino compounds is a daunting task; however, during heating of model systems containing fructose and lysine, 10−36% of the produced HMF derives from the caramelization reactions (*36*). The formation of HMF during caramelization and heating of syrup is influenced primarily by the types and concentrations of sugars and amino compounds, heating regimen and pH. To this end, the HMF mass fractions tend to be higher in the systems comprising reducing sugars, basic amino acids and acidic pH (*37*). The low HMF mass fractions in the maple syrup samples can be attributed to their lower contents of glucose, fructose (p<0.05) and proteins (p<0.05) and higher pH (p<0.05) than of the carob syrups (Table 1 and Table 2), and the mild heating regimen applied in their production (*35*). The higher HMF mass fractions in the date syrup samples than in carob syrup samples might have been caused by their higher mass fractions of glucose and fructose (p<0.05) and lower pH (p<0.05) in view of the similar concentration procedure, entailing open pan evaporation, practiced in the production of carob and date syrups. Pearson’s correlation analysis between HMF mass fractions and pH, sucrose and fructose of the syrups was -6.58 (p<0.01), -0.647 (p<0.01) and 0.510 (p<0.05), respectively. These correlations are in accordance with previous findings from model systems where the formation of HMF was shown to be favoured at acidic pH values due to presence of fructose (*38*). Furthermore, the negative correlation between sucrose and HMF mass fractions is indicative of the lower reactivity of the non-reducing disaccharide sucrose in caramelization and the Maillard-type browning, as reflected in the lower tendency of the high-sucrose containing syrups, and notably the maple syrups, to undergo browning and accumulate HMF (Table 1 and Table 2).

Notwithstanding its beneficial antioxidant activity, HMF has been shown to be carcinogenic and mutagenic and to induce hepato- and nephrotoxicity in laboratory animals, thereby prompting regulatory agencies to set limits to its contents in foods (*38*). Among different approaches to mitigate the contents of HMF in foods, reducing the severity of heat treatment during the concentration step in the production of fruit and tree sap syrups is the most effective. To this end, ~10- and 2.5-fold reduction in HMF mass fractions were achieved upon reducing the temperature during the sap/juice concentration from 100 to 70 °C in the production of date (*39*) and palm sugar syrups (*40*), respectively. The HMF mass fractions of the carob syrup samples included the HMF values reported for Tunisian carob syrup at 450 (*15*) and 1000−2675 mg/kg reported for commercial date syrup produced by open pan evaporation (*39*).

### Sugar composition of carob, date and maple syrups

All carob syrups contained more (p<0.05) glucose and fructose and less sucrose (p<0.05) than maple syrup samples. They also had less glucose and fructose (p<0.05) and, apart from 1 sample that showed no differences (p>0.05), more sucrose (p<0.05) than date syrup (Table 2). The sugar contents of the carob syrups appeared to be intermediate to those of maple syrup that contains almost only sucrose, and date syrup where an invert-sugar-like composition predominates (Table 2). The mass fractions of glucose, fructose and sucrose of the carob syrups included the mass fractions reported for 10 commercial carob syrups from Turkey (*41*). The glucose, fructose and sucrose mass fractions of maple and date syrups were similar to those reported for commercial date (*32*) and maple syrups (*3*). The high sugar mass fraction of carob syrup is responsible for its widespread utilization in the making of a range of ethnic products and its potential use as sugar substitute in the formulation of healthier confections in view of the provision of nutrients and phytochemicals in addition to sweetness. Furthermore, because of its high sugar content, carob syrup has been reported to be a promising substrate for the production of a range of fine chemicals by industrial fermentation (*42*).

### Mineral composition of carob, date and maple syrups

In general, the majority of carob syrups (13−19 samples; 68.4−100%) contained more K (p<0.05), P (p<0.05), Mg (p<0.05) and Na (p<0.05), and less Ca (p<0.05) while the others either did not show differences (p>0.05) (3−6 samples; 16−32%) or contained less (p<0.05) (1−2 samples; 5.3−10.5%) minerals than the maple syrup samples (Table 3). Also, the majority of carob syrups (16−18 samples; 84.2−84.7%) contained more Mg (p<0.05) and Na (p<0.05) and 1−4 samples (5.2−21.1%) contained more (p<0.05) Ca, K and P, while the others either did not show differences (p>0.05) (1−10 samples, 5.3−52.6%) or contained less (p<0.05) (5−10 samples; 26.3−52.6%) minerals than date syrup (Table 3). The relative contents of the investigated minerals in maple syrup were similar to those reported for commercial maple syrups with the highest and lowest contents of K and Na, respectively (*3*). The mineral composition of the date syrup was, in general, within the reported ranges (*10*) (Table 3). These findings indicate that carob syrup is a significant source of K and a good source of Ca, Na, Mg and P and will potentially be a significant contributor to the mineral nutrition of consumers.

**Table 3:** Mineral composition of carob, date and maple syrups

Carob accession	*w*/(mg/100 g)				
P	Ca	Mg	K	Na
Akkar	(116.5±3.0)^a^	(234.28±0.00)^k^	(83.14±4.00)^h^	(1238±34^hi^	(7.9±0.8)^ab^
Selaata	(704±96)^fgh^	(184.3±1.1)^hi^	(46.1±0.8))^cde^	(885±37)^de^	(30.5±1.9)^c^
Wadi El Hojeir	(622±21)^efg^	(170.8±21.4)^gh^	(45.8±8.8))^cde^	(1073±20)^fg^	(39.9±0.4)^e^
Batroun	(536±6)^cde^	(169.8±3.3)^gh^	(59.8±3.0)^g^	(1037±60)^fg^	(43.2±5.5)^e^
Maaroub	(551±10)^def^	(149.1±15.0)^fg^	(42.5±6.4)^cd^	(945±44)^de^	(31.9±5.8)^c^
Bourjin	(606±44)^efg^	(162.6±53.0)^gh^	(41.5±3.9)^cd^	(789±54)^cd^	(40.1±4.9)^e^
Marjayoun	(659±48)^efgh^	(108.1±2.6)^bcd^	(51.6±2.3)^efg^	(1102±48)^g^	(37.6±2.8)^de^
AUB1	(738±106)^gh^	(120.9±12.4)^cdef^	(58.9±4.6)^g^	(975.2±8.2)^ef^	(50.6±3.8)^f^
AUB2	(788±42)^hi^	(97.4±19.9)^abc^	(52.7±7.1)^efg^	(846.8±4.7)^cde^	(29.7±1.7)^c^
AUB3	(791±98)^hi^	(111.5±1.8)^bcde^	(52.2±3.7)^efg^	(952±28)^ef^	(41.6±4.6)^e^
AUB4	(108±27)^i^	(129.0±15.4)^bcd^	(40.3±6.1)^def^	(77998)^i^	(9.5±0.1)^de^
AUB6	(589±32)^efg^	(113.6±11.8)^bcde^	(48.1±2.7)^def^	(970±136)^ef^	(34.2±3.4)^cd^
AUB7	(258±32)^ab^	(77.6±18.5)^a^	(25.6±4.2)^a^	(735±55.^bc^	(12.4±1.4)^b^
AUB8	(631±25)^efg^	(95.3±4.4)^abc^	(37.8±2.1)^bc^	(816±24)^cd^	(49.8±2.3)^f^
AUB9	(908±174)^a^	(110.8±6.9)^def^	(48.2±1.7)^bcd^	(1324±74)^cd^	(37.8±2.6)^ab^
AUB10	(1714±400)^j^	(112.5±0.6)^bcde^	(33.2±0.0)^ab^	(1148±39)^gh^	(94.7±1.9)^h^
AUB11	(626±83)^efg^	(142.4±18.6)^efg^	(52.5±6.5)^efg^	(852.4±4.6)^cde^	(39.2±2.9)^de^
AUB12	(287±12)^b^	(142.4±12.3)^efg^	(55.7±8.4)^fg^	(1536±68)^j^	(29.6±2.9)^c^
AUB14	(409±12)^bcd^	(86.8±7.4)^ab^	(32.4±3.9)^ab^	(631±43)^b^	(79.7±3.3)^g^
Date syrup	(561.3±2.8)^ef^	(166.4±3.2)^gh^	(78.4±0.9)^h^	(1103±168)^g^	(11.5±0.3)^b^
Amber maple syrup	(392±22)^bc^	(220.5±11.7)^jk^	(33.4±0.4)^ab^	(322.2±1.9)^a^	(5.9±1.1)^a^
Dark maple syrup	(120.46±6.08)^a^	(203.1±22.9)^ij^	(32.1±2.0)^ab^	(350±19)^a^	(5.2±0.4)^a^
Range of carob syrups	108−1714	77.6−234.3	25.6−83.1	631−1536	7.9−94.7
Mean±S.D. of carob syrups	613±348	132.6±39.0	47.8±12.5	977±222	38.4±21.0

Mass fractions are expressed on dry mass basis. Values with different letters in superscript in the same column are significantly different (p<0.05) according to Duncan’s multiple range test. AUB1 to AUB14=American University of Beirut

### Total phenols and antioxidant capacity

Apart from 2−4 samples, which did not differ in their TPC (p>0.05), carob syrup samples had higher TPC (p<0.05) than maple syrup samples (Table 4). Additionally, apart from 3 samples that had lower TPC (p<0.05), carob syrup samples had higher (p<0.05) (9 samples, 47.4%) than or did not differ in their TPC (p>0.05) (7 samples, 36.8%) from date syrup (Table 4). Notwithstanding the different solvents used in the extraction of polyphenols from the syrups, carob syrups included the average TPC (expressed as GAE on dry mass basis) of 8 samples from Tunisia (2.1−2.2 g/100 g) (*15*) and 10 samples from Turkey (0.72−1.2 g/100 g) (*41*). The TPC (expressed as GAE on dry mass basis) of maple syrup samples was higher than reported for amber and dark maple syrups at (45.6±18.7) and (72.0±18.2) mg/100 g, respectively (*9*). The high TPC of carob syrups is an added advantage of the use of these syrups as sugar substitutes in the formulation of foods in view of their ability to quench reactive oxygen species and, consequently, to retard/mitigate the development/harmful effects of degenerative diseases (*43*).

**Table 4:** Total phenols and antioxidant capacity of carob, maple and date syrups expressed on dry mass basis

Carob accession	*w*(total phenols as GAE)/(g/100 g)		Antioxidant capacity					
FRAP/(μmol/100 g)	I(g/L)				TE/(mmol/100 g)
Akkar	(728±84)^f^		(2.0±0.0)^efg^	(32.0±2.0)^ab^				(5.6±1.1)^ghi^
Selaata	(732±73)^f^		(1.4±0.3)^bcde^	(16.5±1.5)^ab^				(6.3±0.8)^hi^
Wadi El Hojeir	(1368±40)^g^		(1.65±0.03)^cdef^	(17.0±0.6)^ab^				(5.6±0.6)^ghi^
Batroun	(443±69)^cd^		(0.67±0.06)^ab^	(18.6±0.9)^ab^				(4.2±0.7)^def^
Maaroub	(370±84)^bc^		(1.4±0.5)^bcde^	(46.2±6.8)^b^				(2.5±0.4)^bc^
Bourjin	(781±56)^f^		(2.4±1.0)^fg^	(16.6±1.5)^ab^				(8.3±0.9)^j^
Marjayoun	(358±12)^b^		(1.14±0.08)^bcd^	(26.6±0.9)^ab^				(3.2±0.8)^bcd^
AUB1	(494±15)^de^		(1.13±0.04)^bcd^	(14.4±1.9)^ab^				(4.6±0.1)^efg^
AUB2	(701±62)^f^		(2.68±0.04)^g^	(13.7±0.8)^ab^				(4.9±0.6)^efg^
AUB3	(521±13)^de^		(0.94±0.08)^bcd^	(18.7±0.0)^ab^				(4.4±0.8)^defg^
AUB4	(442±48)^f^		(1.4±0.1)^bcde^	(34.1±0.9)^ab^				(3.6±0.7)^cde^
AUB6	(453±8)^de^		(1.5±0.2)^bcde^	(24.2±1.8)^ab^				(2.3±1.0)^bc^
AUB7	(487.4±1.3)^e^		(1.8±0.3)^def^	(21.1±2.8)^ab^				(4.2±0.2)^def^
AUB8	(345±52)^b^		(0.86±0.02)^abc^	(36.3±0.7)^ab^				(2.5±0.2)^bc^
AUB9	(734±84)^cd^		(1.4±0.8)^bcde^	(20.2±0.2)^ab^				(5.2±0.5)^fgh^
AUB10	(1566.4±2.3)^h^		(2.7±0.4)^g^	(10.1±1.3)^ab^				(6.6±0.7)^i^
AUB11	(336±38)^ab^		(1.02±0.08)^bcd^	(36.3±1.0)^ab^				(2.4±0.2)^bc^
AUB12	(2215±6)^i^		(14.9±1.4)^h^	(4.9±0.7)^a^				(20.5±2.2)^k^
AUB14	(741±17)^f^		(2.4±0.8)^fg^	(19.9±0.0)^ab^				(5.6±0.5)^ghi^
Date syrup	(461±13)^de^		(1.06±0.02)^bcd^	(33.7±0.6)^ab^				(2.1±0.0)^b^
Amber maple syrup	(261.0±2.6)^a^		(0.10±0.00)^a^	(647±14)^c^				(0.4±0.0)^a^
Dark maple syrup	(314.0±2.3)^ab^		(0.11±0.00)^a^	(501±59)^d^				(0.4±0.0)^a^
Range of carob syrups	336−2214	0.7−14.9			4.9−46.2	2.3−20.5		
Mean±S.D. of carob syrups	729±487	2.3±3.1			23.0±10.8		5.4±4.0	

GAE=gallic acid equivalents, TE=Trolox equivalents, FRAP=ferric reducing antioxidant power, TEAC=Trolox equivalent antioxidant capacity, DPPH=2,2-diphenyl-1-picrylhydrazyl. Values with different letters in superscript in the same column are significantly different (p<0.05) according to Duncan’s multiple range test. AUB1 to AUB14=American University of Beirut

Apart from 5 samples that did not differ (p>0.05) from maple syrup, all carob syrups had higher antioxidant capacity (p<0.05), as determined by the FRAP assay, than maple syrup (Table 4). Moreover, 14 carob syrup samples (73.7%) did not differ (p>0.05), while 5 samples (26.3%) showed higher antioxidant capacity (p<0.05), according to the FRAP assay, than date syrup (Table 4).

All carob syrups showed higher antioxidant capacity (p<0.05), as determined by the TEAC assay, than the maple syrup samples and, apart from 4 samples (21.1%) that did not show differences (p>0.05), the carob syrup samples had higher antioxidant capacity (p<0.05) than the date syrup (Table 4).

All carob syrups showed higher antioxidant capacity (p<0.05), as determined by the DPPH assay, than the maple syrup samples. Also, 3 carob syrup samples had higher antioxidant capacity (p<0.05), while the others did not show differences in their antioxidant capacity (p>0.05) from that of the date syrup (Table 4). The carob syrups had higher mean antioxidant capacity (lower IC_50_) than that reported for carob syrup at 47.2 mg/L (*13*). For comparison, ascorbic acid showed an IC_50_ of (74±5.7) mg/L under the same conditions in the present work. The IC_50_ values of maple syrup (647.1 and 501 mg/L) in DPPH assay were higher than the IC_50_ values of their ethanol extracts at 97.6−102.4 mg/L (*44*).

Notwithstanding the differences in the assay protocols and the use of extracts or whole syrups in the determinations, carob syrups have been reported to contain significant quantities of total phenols and to exhibit high antioxidant capacity, comparable to that of butylated hydroxytoluene, in several *in vitro* antioxidant capacity assays (*13*, *15*, *41*). Similar findings have been reported for the total phenols and antioxidant capacity of maple (*1*, *9*, *44*) and date syrups (*45*). However, when analyzed under the same conditions, the vast majority of carob syrup samples had higher TPC than amber and dark maple syrup and comparable or higher TPC than date syrup (Table 4). Similar patterns were observed for the antioxidant capacity with most of the carob syrup samples exhibiting higher antioxidant capacity than amber and dark maple syrup and comparable or higher than date syrup in the ABTS, DPPH and FRAP assays (Table 4).

**Fig. 1:**
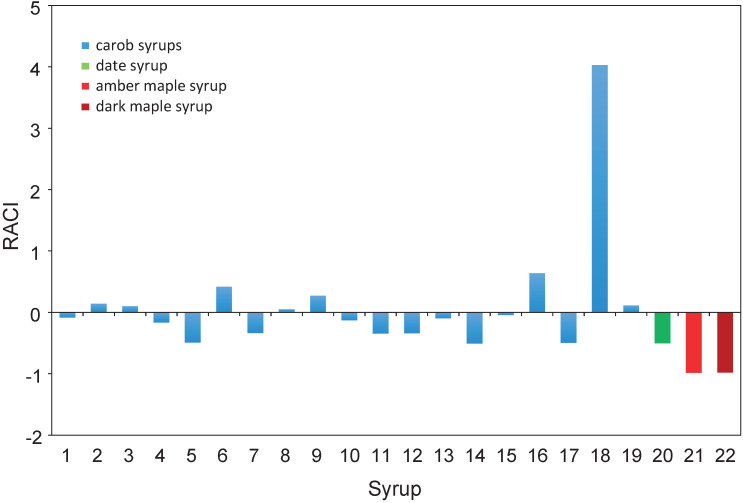
Relative antioxidant capacity indices (RACI) of the analyzed syrups (carob syrups prepared from the carob accessions as listed in Tables 1−5)

The antioxidant potential of foods is frequently determined by a combination of several tests that are based on different principles and expressed in different units. Accordingly, indices that integrate the data from different antioxidant assays are often utilized to construct measures of the total antioxidant capacity of foods (*46*). To this end, the data from the different antioxidant tests were normalized and the derived z-scores were averaged to generate relative antioxidant capacity indices (RACI) (*46*) for the different syrups (Fig. 1). All the carob syrups exhibited higher RACI than maple syrup and, apart from 3 carob syrups that had close RACI, higher than date syrup (Fig. 1). The TPC, as determined by the Folin-Ciocalteu reagent, correlated strongly with the RACI (r=0.881, p<0.01), thereby offering further support to the modulation of the antioxidant capacity of foods and biological materials by their endogenous phenolics (*47*).

Given the pivotal role of phenols and their associated antioxidant potential in combating the development and progression of degenerative diseases and mitigating their ill effects (*43*), the present findings indicate the superiority of carob syrup as a functional dietary ingredient as compared to date and maple syrups. The reported higher anti-inflammatory and antimutagenic effects of carob syrup than those of cane, grape and sorghum molasses (*14*) further attest to its advanced health-promoting effects relative to those of cereal, fruit and tree sap syrups.

**Fig. 2:**
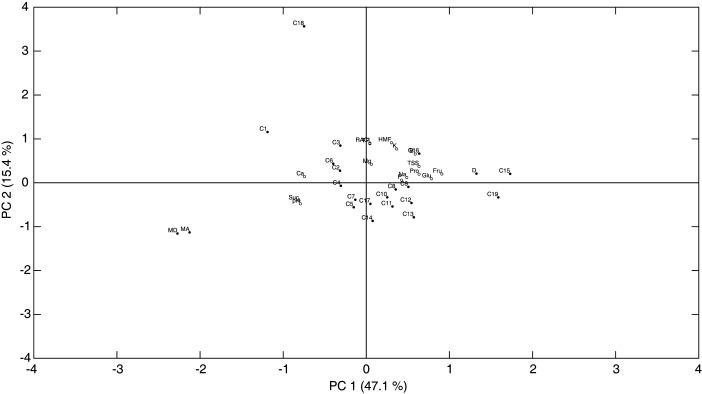
Principal component analysis biplot showing relationships amongst the chemical and physicochemical parameters and the analyzed syrups (C1-C19 refer to the carob syrups prepared from the carob accessions as listed in Tables 1−5). Fru=fructose, Glu=glucose, Suc=sucrose, HMF=5-hydroxymethylfurfural, Pro=proteins, RACI=relative antioxidant capacity, TP=total phenols, D=date syrup, MA=maple syrup amber, MD=maple syrup dark

The acquired data were subjected to principal component analysis (PCA) to discern the variables that are operative in mediating the relationships amongst the syrups and to identify possible groupings of the investigated samples. Two principal components accounting for 62.5% of the total variance separated the syrups into distinct groups (Fig. 2). The first principal component (PC1), which explained 47.1% of the total variance, related chiefly to the sugars (glucose, fructose and sucrose), proteins, pH, TSS, BI and Ca, while the second principal component (PC2) was associated mainly with the BI, HMF, TP, RACI and K. It is noteworthy that the reactants, conditions, the index of caramelization and the Maillard-type of browning loaded on PC1. To this end, fructose, glucose, proteins and BI loaded on the positive side of PC1, thereby confirming the direct relationship between browning intensity and these reactants, while the pH and sucrose loaded heavily on the negative side of PC1 in accord with the intensification of browning at acid pH values and the sluggish reactivity of sucrose in browning under the conditions of syrup production. The TP, RACI, HMF and BI loaded on the positive side of PC2, thereby suggesting that this principal component is mainly associated with antioxidant capacity of the syrups given the high correlation of the TPC and antioxidant capacity with the reported antioxidant properties of HMF (*38*). While the carob syrups showed a diffuse pattern in sugar composition, the date and maple syrups loaded heavily on the opposite sides of PC1 in congruence with the invert-sugar-like composition of the former and the almost exclusive presence of sucrose in the latter. All the carob syrups loaded higher than the maple syrups on PC2, which is indicative of their higher TPC, HMF and antioxidant capacity levels; however, the date syrup loaded amongst the carob syrups on PC2 in accord with its TPC, HMF and antioxidant capacity levels being within the ranges of these parameters exhibited by the carob syrup samples.

### Phenolic profiles of carob, date and maple syrups

Most carob syrup samples contained more (p<0.05) phenolic acids (caffeic, *t*-cinnamic, *p*-coumaric, gallic and syringic acids) with few showing no differences in their phenolic acid contents from amber and dark maple syrups (Table 5). In the analyzed flavonoids, all carob syrup samples contained less catechin (p<0.05), while most did not show differences (7−15 samples, p>0.05) or contained more (4−12 samples, p<0.05) quercetin than maple syrup (Table 5). Furthermore, all carob syrup samples contained less (p<0.05) caffeic acid, more (p<0.05) *p*-coumaric, syringic and *t*-cinnamic acids, and, apart from 8 samples that did not show differences (p>0.05), more gallic acid than date syrup (Table 5). Moreover, apart from 7 and 8 samples that did not exhibit differences (p>0.05), carob syrup samples contained more (p<0.05) catechin and quercetin than date syrup (Table 5). Of the analyzed phenolics, gallic acid correlated with TPC (r=0.864, p<0.01) and RACI (r=0.972, p<0.01), quercetin with TPC (r=0.586, p<0.01) and RACI (r=0.621, p<0.01) and catechin with RACI (r=-0.490, p<0.05), thereby suggesting that these phenolics are the most operative in shaping the TPC and antioxidant capacity of the investigated syrups.

**Table 5:** Mass fractions of phenolic acids and flavonoids of carob, maple and date syrups expressed on dry mass basis

Carob accession	*w*/(μg/g)						
Gallic acid	Syringic acid	Caffeic acid	*t*-cinnamic acid	*p*-coumaric acid	Catechin	Quercetin
Akkar	(4266±6)^cdef^	(1.2±0.3)^f^	(0.9±0.2)^bcdef^	(14.2±1.0)^de^	(13.3±0.8)^hi^	(1.0±0.1)^de^	(1.21±0.01)^abc^
Selaata	(770±46)1^j^	(0.08±0.02)^a^	(1.01±0.1)^cdefg^	(17.6±2.7)^gh^	(12.9±2.2)^gh^	(1.05±0.03)^de^	(1.31±0.05)^cde^
Wadi El Hojeir	(1377±91)^gh^	(1.93±0.01)^hi^	(1.3±0.1)9^h^	(15.0±0.8)^ef^	(11.0±1.0)^efgh^	nd^a^	(1.56±0.00)^f^
Batroun	(204±19)^ab^	(0.92±0.06)^def^	(0.72±0.03)^b^	(15.5±2.8)^efg^	(9.8±1.1)^cdef^	(1.28±0.03)^de^	(1.3±0.2)^cde^
Maaroub	(565±7)^fg^	(1.67±0.05)^gh^	(0.78±0.06)^bc^	(13.7±0.5)^de^	(8.8±0.5)^cde^	(1.6±0.3)^f^	(1.69±0.03)^f^
Bourjin	(2351±333)^l^	(0.41±0.08)^bc^	(1.12±0.01)^fgh^	(8.1±0.9)^bc^	(15.34±0.02)^ij^	(0.19±0.01)^a^	(1.23±0.02)^abcd^
Marjayoun	(523±15)^efg^	(0.34±0.09)^abc^	(1.04±0.01)^defgh^	(8.4±0.2)^bc^	(8.2±1.4)^bcd^	(0.63±0.08)^bc^	(1.23±0.01)^abcd^
AUB1	(828±76)^hi^	(0.90±0.06)^de^	nd^a^	(10.4±1.2)^c^	(13.3±0.8)^hi^	(1.0±0.1)^de^	(1.32±0.03)^cde^
AUB2	(955±67)^i^	(1.75±0.30)^ghi^	nd^a^	(7.7±0.5)^b^	(10.62±0.07)^efg^	(0.31±0.07)^ab^	(1.29±0.04)^bcde^
AUB3	(657±84)^bcdef^	(0.63±0.02)^cd^	(1.1±0.1)^fgh^	(8.1±1.7)^bc^	(15.9±1.2)^j^	(0.88±0.08)^cde^	(1.19±0.06)^ab^
AUB4	(394±24)^hi^	nd^j^	(0.8±0.1)^defgh^	(18.3±0.8)^fgh^	(25.4±0.1)^def^	(0.07±0.02)^a^	(1.25±0.02)^abcd^
AUB6	(489±61)^cdefg^	(0.8±0.2)^de^	(0.89±0.09)^bcdef^	(23.5±0.4)^j^	(11.8±1.2)^fgh^	(1.0±0.1)^de^	(1.55±0.04)^f^
AUB7	(659±21)^gh^	(1.6±0.5)^g^	(0.77±0.09)^bc^	(19.88±0.01)^i^	(12.9±1.3)^gh^	(1.1±0.2)^de^	(1.35±0.06)^e^
AUB8	(361±24)^abcd^	(2.0±0.3)^ij^	(1.06±0.09)^efgh^	(29.0±2.1)^k^	(15.7±0.8)^j^	(1.0±0.1)^de^	(1.17±0.05)^ab^
AUB9	(838±27)^bcde^	(2.3±0.2)^a^	(1.0±0.1)^bcd^	(16.6±1.5)^hi^	(9.9±1.4)^l^	nd^a^	(1.23±0.02)^abcde^
AUB10	(1891±186)^k^	(1.1±0.2)^ef^	(0.8±0.1)^bcde^	(12.6±0.9)^d^	(7.6±0.8)^bc^	(1.2±0.2)^e^	(1.31±0.02)^cde^
AUB11	(293±70)^hi^	(0.16±0.07)^ab^	(0.77±0.02)^bc^	(22.6±2.5)^j^	(17.6±1.8)^jk^	(0.8±0.2)^cd^	(1.15±0.07)^a^
AUB12	(7666±370)^m^	(2.02±0.07)^ij^	(0.89±0.01)^bcdef^	(8.8±0.6)^bc^	(6.0±1.1)^b^	nd^a^	(1.84±0.08)^g^
AUB14	(948±30)^i^	(1.6±0.2)^gh^	(1.2±0.2)^gh^	(15.0±1.3)^ef^	(18.5±3.7)^k^	(0.33±0.07)^ab^	(1.33±0.08)^de^
Date syrup	(254±17)^abc^	nd^a^	(7.7±0.4)^i^	nd^a^	(2.8±0.2)^a^	(0.14±0.02)^a^	(1.15±0.03)^a^
Amber maple syrup	(135±26)^a^	nd^a^	nd^a^	nd^a^	(0.71±0.08)^a^	(2.6±0.3)^g^	(1.23±0.02)^abcd^
Dark maple syrup	(221±86)^abc^	nd^a^	nd^a^	nd^a^	(0.9±0.1)^a^	(1.75±0.03)^f^	(1.25±0.03)^abcde^
Range of carob syrups	204-7666	nd-2.26	nd-1.26	7.7-29.0	6.0-25.4	nd-1.57	1.2-1.8
Mean±S.D. of carob syrups	1166±1666	1.1±0.7	0.8±0.3	15.0±5.9	12.9±4.6	0.7±0.5	1.3±0.2

Values with different letters in superscript in the same column are significantly different (p<0.05) according to Duncan’s multiple range test. dm=dry mass, nd=not detected. AUB1 to AUB14=American University of Beirut

The phenolic profiles of fruit and tree sap syrups are shaped by the liquid-liquid extraction protocol, use of resins or solid-phase extraction to remove interfering compounds, and the chromatographic conditions employed in the identification and quantification of the individual phenolics. Accordingly, only broad comparisons of phenolic profiles of syrups reported by different authors are plausible. Under the analytical conditions used in this work, gallic acid was the major phenolic in carob, date and maple syrups (Table 5). The preponderance of gallic acid in carob syrup has been attributed to its preferential extraction and release to other gallic acid-containing phenolics in the kibbles during syrup preparation (*48*). Furthermore, notwithstanding the differences in analytical protocols, the phenolic patterns of the carob syrups obtained in the present work were, in general, comparable to those reported elsewhere (*13*). The predominance of gallic acid in the date syrup may be related to it being the main phenol in different varieties and clones of dates (*49*). In contrast to the present findings, protocatechuic acid, coniferyl alcohol and vanillin were the major phenolics in methanol extracts of maple syrup prepared by solid-phase extraction (*50*).

## CONCLUSIONS

Laboratory-made carob syrups contained more proteins than date and maple syrups and more minerals (Ca, Mg, Na, K and P) than maple syrup and, in general, higher or at least similar mass fractions of the indicated minerals than date syrup. The carob syrups had sugar compositions intermediate to those of the invert-sugar-like composition of date syrup and the overwhelmingly sucrose-containing maple syrup. Furthermore, the vast majority of carob syrups contained more total phenols than maple syrup and higher, or at least comparable, levels of total phenols than date syrup. Similar patterns to those of total phenols were found for the antioxidant capacity of the syrups by *in vitro* antioxidant capacity assays. Gallic acid was the major phenolic acid in the carob, date and maple syrups, and, in general, the carob syrups contained more phenolic acids and flavonoids than the other syrups. The carob syrups browned more and generated more hydroxymethylfurfural than maple syrup during the heat concentration step and, in general, were less brown and contained lower mass fractions of hydroxymethylfurfural than date syrup. These findings indicate that carob syrup exhibits a favourable phenolic profile, provides more proteins and minerals, contains more total phenols and has a higher antioxidant potential than maple and date syrups. These traits render carob syrups strong candidates for the category of mainstream syrups in international trade with obvious economic returns to the largely least developed carob-producing countries. However, the age-old practice of heat concentration in open vats leads to excessive browning and the concomitant formation of high levels of the potentially mutagenic and carcinogenic hydroxymethylfurfural and, therefore, milder heating regimens are warranted in the production of carob syrups.

## Figures and Tables

**Table S1:** Morphological characteristics of carob pods from the accessions used in molasses making

Carob accession	*m*/g	*l*/cm	*b*/cm	*δ*/cm	*N*(seed)
Akkar	(23.8±5.2)^g^	(18.5±2.9)^h^	(3.0±2.1)^ab^	(0.9±0.2)^defg^	(11.0±1.5)^hij^
Selaata	(19.4±3.7)^ef^	(13.2±1.7)^cde^	(2.3±0.2)^a^	(0.8±0.2)^bcd^	(9.0±1.1)^cdef^
Wadi El Hojeir	(11.7±2.1)^abc^	(15.3±0.7)^efg^	(1.7±0.1)^a^	(0.6±0.1)^a^	(12.0±1.9)^j^
Batroun	(18.1±3.6)^def^	(11.0±1.3)^ab^	(2.3±0.2)^a^	(1.0±0.2)^efg^	(11.0±1.6)^hij^
Maaroub	(15.7±1.8)^cde^	(10.1±0.5)^a^	(1.9±0.1)^a^	(0.8±0.3)^bcde^	(9.0±1.4)^bcd^
Bourjin	(19.2±5.8)^ef^	(14.2±2.3)^def^	(2.4±0.2)^ab^	(1.0±0.2)^defg^	(11.0±1.3)^ghij^
Marjayoun	(15.6±2.4)^cde^	(10.0±0.6)^a^	(1.7±0.3)^a^	(0.9±0.1)^defg^	(8.0±1.2)^ab^
AUB1	(21.1±3.6)^fg^	(17.1±2.2)^gh^	(2.2±0.3)^a^	(0.8±0.1)^bcd^	(10.0±0.5)^fghij^
AUB2	(12.8±4.8)^abc^	(13.2±2.5)^cde^	(2.2±0.2)^a^	(0.7±0.1)^abc^	(13.0±0.5)^k^
AUB3	(25.1±5.6)^g^	(15.9±4.0)^fg^	(2.2±0.4)^a^	(0.9±0.1)^defg^	(11.0±0.5)^hij^
AUB4	(18.1±4.0)^a^	(10.2±2.9)^abc^	(2.3±0.4)^a^	(0.7±0.1)^ab^	(8.0±1.1)^bc^
AUB6	(11.6±2.2)^ab^	(9.9±1.8)^a^	(2.0±0.3)^a^	(0.9±0.2)^defg^	(9.0±2.3)^cdefg^
AUB7	(14.5±4.4)^bcd^	(14.6±2.1)^def^	(3.8±5.9)^b^	(0.9±0.2)^cdefg^	(11.0±1.1)^hij^
AUB8	(18.1±4.1)^def^	(10.1±1.4)^a^	(2.2±0.3)^a^	(1.0±0.1)^g^	(10.0±0.9)^efghi^
AUB9	(25.3±8.1)^g^	(11.3±2.1)^de^	(1.7±0.3)^ab^	(0.7±0.1)^defg^	(8.0±0.6)^cde^
AUB10	(11.8±3.3)^abc^	(12.7±1.9)^bcd^	(1.9±0.3)^a^	(1.0±0.2)^efg^	(10.0±1.5)^defgh^
AUB11	(22.3±4.8)^fg^	(10.3±1.9)^a^	(2.3±0.3)^a^	(0.9±0.2)^cdefg^	(7.0±0.9)^a^
AUB12	(10.5±2.6)^ab^	(12.5±4.0)^bcd^	(1.9±0.2)^a^	(0.9±0.4)^bcdef^	(11.0±1.0)^ij^
AUB14	(19.2±4.7)^g^	(11.6±0.2)^a^	(2.1±0.6)^a^	(0.9±0.0)^fg^	(15.0±1.3)^l^

Values with different letters in superscript in the same column are statistically different (p<0.05) according to Duncan’s multiple range test. AUB1 to AUB14=American University of Beirut
